# Tanshinone Content Prediction and Geographical Origin Classification of *Salvia miltiorrhiza* by Combining Hyperspectral Imaging with Chemometrics

**DOI:** 10.3390/foods13223673

**Published:** 2024-11-18

**Authors:** Yaoyao Dai, Binbin Yan, Feng Xiong, Ruibin Bai, Siman Wang, Lanping Guo, Jian Yang

**Affiliations:** 1 State Key Laboratory for Quality Ensurance and Sustainable Use of Dao-di Herbs, National Resource Center for Chinese Materia Medica, China Academy of Chinese Medical Sciences, Beijing 100700, China; daiyaoyao@stu.cdutcm.edu.cn (Y.D.); yb51598@126.com (B.Y.); xfzyzy@163.com (F.X.); bairuibin2022@163.com (R.B.); wsm8192023@163.com (S.W.); glp01@126.com (L.G.); 2 School of Pharmacy, Chengdu University of Traditional Chinese Medicine, Chengdu 610072, China; 3 Key Laboratory of Biology and Cultivation of Herb Medicine, Ministry of Agriculture and Rural Affairs, Beijing 100700, China; 4 Dexing Research and Training Center of Chinese Medical Sciences, Dexing 334213, China

**Keywords:** *Salvia miltiorrhiza*, hyperspectral imaging, chemometrics, traceability, content prediction

## Abstract

Hyperspectral imaging (HSI) technology was combined with chemometrics to achieve rapid determination of tanshinone contents in *Salvia miltiorrhiza*, as well as the rapid identification of its origins. Derivative (D1), second derivative (D2), Savitzky–Golay filtering (SG), multiplicative scatter correction (MSC), and standard normal variate transformation (SNV) were utilized to preprocess original spectrum (ORI). Partial least squares discriminant analysis (PLS-DA) and support vector machine (SVM) models were employed to discriminate 420 *Salvia miltiorrhiza* samples collected from Shandong, Hebei, Shanxi, Sichuan, and Anhui Provinces. The contents of tanshinone IIA, tanshinone I, cryptotanshinone, and total tanshinones in *Salvia miltiorrhiza* were predicted by the back-propagation neural network (BPNN), partial least square regression (PLSR), and random forest (RF). Finally, effective wavelengths were selected using the successive projections algorithm (SPA) and variable iterative space shrinkage approach (VISSA). The results indicated that the D1-PLS-DA model performed the best with a classification accuracy of 98.97%. SG-BPNN achieved the best prediction effect for cryptotanshinone (RMSEP = 0.527, RPD = 3.25), ORI-BPNN achieved the best prediction effect for tanshinone IIA (RMSEP = 0.332, RPD = 3.34), MSC-PLSR achieved the best prediction effect for tanshinone I (RMSEP = 0.110, RPD = 4.03), and SNV-BPNN achieved the best prediction effect for total tanshinones (RMSEP = 0.759, RPD = 4.01). When using the SPA and VISSA, the number of wavelengths was reduced below 60 and 150, respectively, and the performance of the models was all very good (RPD > 3). Therefore, the combination of HSI with chemometrics provides a promising method for predicting the active ingredients of *Salvia miltiorrhiza* and identifying its geographical origins.

## 1. Introduction

*Salvia miltiorrhiza* Bunge is an herb of the genus *Salvia*, family *Lamiaceae*, which is widely distributed in China, such as in provinces of Shandong, Sichuan, Anhui, Hebei, and Shanxi [1,2]. Its dried roots and rhizomes, called “Danshen” in China, are a well-known traditional Chinese medicine, with about 80 million tons used annually. It is commonly used to treat chest paralysis, heart pain, and menstrual disorders. The powder of *S. miltiorrhiza* is also a common health food used in soups, tea, or beverages [3]. Lipid-soluble tanshinones such as tanshinone I (Tan I), tanshinone IIA (Tan IIA), and cryptotanshinone (CTS) are the main active compounds of *S. miltiorrhiza* [4], which had been indicated to have antitumor [5], osteoporosis-inhibiting [6], anti-inflammatory [7], anti-anxiety [8], and antithrombotic [9] effects. Furthermore, the sum of Tan I, Tan IIA, and CTS was employed as an important indicator for evaluating the quality of *S. miltiorrhiza* [10]. The quality of *S. miltiorrhiza* varied among the geographical origins due to the different environmental conditions [2]. For example, *S. miltiorrhiza* from Shandong has a higher content of tanshinones [11]. This has led to price differences in *S. miltiorrhiza* from different origins and sometimes to fraud by sellers claiming false areas of origin. Therefore, a rapid method to access the active compound’s content and distinguish the geographical origin of *S. miltiorrhiza* is urgently needed.

The traditional methods for determining tanshinones in *S. miltiorrhiza* include high-performance liquid chromatography (HPLC) [12], ultra-performance liquid chromatography-mass spectrometry/mass spectrometry (UPLC-MS/MS) [13], and ultra-performance liquid chromatography-triple quadrupole mass spectrometry (UPLC-QQQ-MS) [14]. The traditional methods for identifying the origins of herbs include inductively coupled plasma mass spectrometry (ICP-MS) [15], isotope ratio mass spectrometry (IRMS) [16], HPLC [17], etc. Although these methods have the advantages of high accuracy, they also have the disadvantages of being destructive, time-consuming, and costly [18]. Therefore, the quality evaluation and origin tracing of *S. miltiorrhiza* require a non-destructive, fast, and green technical method.

Hyperspectral imaging (HSI) technology has the advantages of being fast, non-destructive, and green [19]. It can be applied to content prediction, origin tracing, and authenticity identification of traditional Chinese medicines [20]. Compared with infrared and ultraviolet imaging, HSI adds one-dimensional spectral data to normal two-dimensional spatial images to form three-dimensional data [21]. This results in massive hyperspectral data and a large amount of redundant information. HSI combined with chemometrics can provide a more comprehensive quality evaluation and origin identification of traditional Chinese medicine. Liu et al. [22] combined the HSI technique with partial least square regression (PLSR), support vector regression (SVR), and random forest regression (RFR) algorithms to establish a quantitative model. The model could predict the content of Lycium barbarum polysaccharide, total flavonoid, betaine, and vitamin C in Lycium barbarum. Wang et al. predicted the content of ginsenoside Rg2 in ginseng by combining HSI and chemometrics [23]. Liu et al., identified the origin of hawthorn based on hyperspectral [24]. However, there is limited research on origin identification and content prediction of tanshinones in *S. miltiorrhiza* using HSI.

This study aimed to combine HSI with chemometrics to predict the content of tanshinones in *S. miltiorrhiza* and to identify their geographical origins. The specific objectives were (1) to investigate the differences in the active compounds in *S. miltiorrhiza* and to identify *S. miltiorrhiza* from different origins using HSI in combination with chemometrics; (2) to predict the contents of CTS, Tan IIA, and Tan I using HSI and compare the noise reduction methods and the regression models; and (3) to improve the performance of the model by selecting the wavelengths of HSI using the successive projections algorithm (SPA) and variable iterative space shrinkage approach (VISSA).

## 2. Materials and Methods

### 2.1. Sample Collection and Pretreatment

Samples of *S. miltiorrhiza* were collected from October to December 2021 from thirty cultivated lands in five provinces (Shandong, Hebei, Shanxi, Sichuan, and Anhui) in China (Supplementary Table S1). Fourteen healthy and uniformly growing plants were randomly dug in each cultivated land using the checkerboard sampling method (84 samples per province, 420 samples in total). All samples were identified as the dried roots and rhizomes of *S. miltiorrhiza* Bge. of the family Lamiaceae by Associate Researcher Yang Jian from the National Resource Center for Chinese Materia Medica, China Academy of Chinese Medical Sciences. The roots and rhizomes of the plants were air-dried and ground into fine powder. Then, the powder samples were sieved through a 65-mesh sieve for spectral acquisition.

### 2.2. Data Collection Using an HSI System and Extraction of Interest Origins

Spectral information of 420 samples of *S. miltiorrhiza* was obtained using a visible and short-wave/long-wave near-infrared hyperspectral imaging spectroscopy system (VIS-NIR-HSI, HySpex VNIR-1800/HySpex SWIR 384, Norsk Elektro Optikk, Oslo, Norway). The system consisted of visible near-infrared (VNIR) and short-wave/long-wave near-infrared (SWIR) lenses, two 150 W tungsten halogen lamps (H-LAM, Norsk Elektro Optikk, Oslo, Norway), a conveyor belt, and a computer for data collection. The VNIR spectral range was 410~990 nm, each wavelength interval was 5.4 nm, and a total of 108 wavelengths were obtained. The SWIR spectral range was 950~2500 nm, each wavelength interval was 5.45 nm, and a total of 288 wavelengths were obtained. Then, 2 g of *S. miltiorrhiza* powder was placed in a 3.5 cm disposable Petri dish, which was shaken gently until the surface was evenly distributed. The exposure times for VNIR and SWIR were 0.0035 s and 0.0045 s, respectively. The samples were placed 30 cm from the lens. The speed of the conveyor belt was 2.5 mm/s.

To attenuate the noise interference due to the dark current and the uneven distribution of the light source in the HSI acquisition process, black and white plate correction of the HSI data was performed before further analysis. The correction formula is as follows:

(1) $$R=\frac{R_{r}{\;-\;R}_{b}}{R_{w}{\;-\;R}_{b}}$$

where *R* denotes the corrected spectral data, *R_r_* denotes the original spectral data, *R_b_* represents the blackboard reference data obtained by turning off the light and blocking the camera lenses, and *R_w_* refers to the whiteboard reference data obtained from the whiteboard with a reflectivity of 99% [25]. The spectral information of each powder sample of *S. miltiorrhiza* was treated as one origin of interest (ROI) and extracted using the ENVI 5.3 software (Harris Geospatial Solutions Inc., Boulder, CO, USA).

### 2.3. Chemometrics Analysis

#### 2.3.1. Pretreatment

Spectral data contain not only information related to the composition of the sample but also interference information such as electrical noise, matrix background, and scattering effects. To reduce the influence of interference information and improve the accuracy and reliability of the models, the original spectrum (ORI) was preprocessed in this study using five methods, namely derivative (D1), second derivative (D2), Savitzky–Golay filtering (SG), multiplicative scatter correction (MSC), and standard normal variate transformation (SNV).

Specifically, D1 displays the rate of change in light intensity as a function of wavelength. D2 displays local slope information for spectral data. They could correct the baseline and highlight the peak and absorption features of the spectral data [26]. The SG is commonly employed to eliminate noise. It smooths the data by fitting a polynomial over the data and calculating the value at each point [27]. MSC is widely used to correct scattering effects in spectral data to reflect the true chemical information of the sample [28]. Additionally, SNV is commonly utilized to correct additive and multiplicative information in spectral data and thus improve the interpretability of spectral data [29].

#### 2.3.2. Classification Models

Two methods, partial least squares discriminant analysis (PLS-DA) and support vector machines (SVMs), were used in this study to establish the classification models.

PLS-DA is a supervised linear algorithm developed based on the least squares algorithm, and it is commonly used as a qualitative analysis method in spectral analysis. It establishes a qualitative analysis model based on the characteristic parameters of the known sample set [18], and then it can judge unknown samples. In this study, the characteristic parameters were set at 4~15.

The SVM is a classical supervised learning algorithm. Its core idea is to find a hyperplane in feature space that makes the distance between the samples on either side of the plane as large as possible. So, the generalization of the model is improved. The algorithm can be applied to non-linear classification, high-dimensional data mining, and regression fitting. Also, it is suitable for the classification of small- and medium-sized complex datasets [30].

#### 2.3.3. Prediction Models

Three methods, the back-propagation neural network (BPNN), PLSR, and RF, were employed in this study to establish the content prediction models.

The BPNN is a multilayer feedforward neural network trained by an error back-propagation algorithm. It consists of the input layer, hidden layer, and output layer. Each layer contains multiple nodes. The number of neuron nodes in the input and output layers is determined. The number of neuron nodes in the hidden layer can be changed, and this has a large impact on the prediction results [31].

PLSR is a linear regression method which integrates the advantages of principal component analysis, typical correlation analysis, and multiple linear regression. It can be utilized to solve the problem of multicollinearity of variables and the problem of predicting variables with small sample sizes [32].

RF is an integrated learning algorithm that can be employed to solve classification, regression, and clustering problems for large sample sizes. It consists of classification decision trees. To make a prediction a sample, the random forest obtains the final result by averaging or voting on the predicted values of all the decision trees [33].

#### 2.3.4. Effective Wavelength Screening Algorithms

HSI with many bands and high data dimensions may lead to redundant interference problems, thus increasing the computational burden of modeling. Screening effective wavelengths is a common method to simplify the models and improve the calculation speed of the models. In this study, the SPA and VISSA were adopted to select the effective wavelengths.

The SPA is a forward feature variable selection method. It uses vector projection analysis and correction models to select effective wavelengths with low redundancy and low covariance that can reflect the key information of the sample spectrum [34].

The VISSA is a new algorithm for screening effective wavelengths based on model cluster analysis strategy (MCA) and weighted binary matrix sampling (WBMS). It can eliminate irrelevant variables in each iteration while preserving the key variables, thereby obtaining the optimal combination of variables [20].

### 2.4. Reference Determination of Four Tanshinone Content

The total tanshinones were the sum of CTS, Tan IIA, and Tan I content. In this study, according to the established method, which was based on the previous published method in our study group [35], CTS, Tan IIA, and Tan I contents were determined by ultra-performance liquid chromatography-quadrupole/trap-mass spectrometry (UPLC-QTRAP-MS/MS-6500, ABSCIEX, Framingham, MA, USA). Specifically, 15 mg of sample powder was placed into a 2 mL centrifuge tube and extracted with 1.5 mL of methanol solution (LC/MS grade, Fisher Scientific, Hampton, NH, USA) for 40 min using an ultrasonic bath (250 W, 40 kHz). Then, centrifugation (12,000 rpm) was performed for 5 min, and the supernatant was obtained through a 0.22 µm filter membrane. The chromatographic column was an ACQUITY UPLC BEH C18 column (2.1 mm × 100 mm, 1.7 µm, Waters, Milford, MA, USA) with a column temperature of 40 °C, an injection volume of 1 µL, and a flow rate of 0.5 mL·min^−1^. The mobile phases were acetonitrile (LC/MS grade, Fisher Scientific, Hampton, NH, USA) with 0.1% formic acid (LC/MS grade, Fisher Scientific, Hampton, NH, USA) (A) and water with 0.1% formic acid (B). The elution gradients were as follows: 0~3.5 min, 65%~75% A; 3.5~4.5 min, 75%~65% A; and 4.5~6.5 min, 65% A.

Mass spectrometry analyses were conducted in the following conditions. The electrospray ionization source (ESI) mode was configured with positive ion scanning and an ion source temperature of 550 °C. The curtain air (CUR) flow rate was 30 L/min. The spray voltage was 5500 V. The atomizing gas flow rate was 50 L/min. The heating assist gas flow rate was 50 L/min, and the acquisition method was the multi-response monitoring mode (MRM). The conditions of Q1 MASS, Q3 MASS, depolymerization voltage, collision energy, and injection voltage were optimized using standard samples of CTS (CAS: 20090909; Beit Renkang, Beijing, China), Tan IIA (CAS: 22012016; Beit Renkang, Beijing, China), and Tan I (CAS: 20090901; Beit Renkang, Beijing, China). A quantitative regression equation was formulated based on the peak area (Y) and the corresponding concentration (x) of the standard samples. The coefficient of determination was 0.992 5~0.999 9. In the precision test, stability test, repeatability test and recovery test, the RSD of CTS, Tan IIA, and Tan I was 0.64%~2.70%, 2.60%~3.90%, 1.30%~3.30%, and 1.60%~11.00%. The total ion current diagrams of a *Salvia miltiorrhiza* sample are in Supplementary Figure S1.

### 2.5. Statistical Analysis

Significant differences (*p* < 0.05) in tanshinones content of *S. miltiorrhiza* from different origins were analyzed using the R software (version 4.2.0) with Kruskal–Wallis by adopting the “Games–Howell” multiple comparison method (“userfriendlyscience” package), considering the non-normality of the data. Meanwhile, MATLAB R2024a was used for establishing models and drawing figures.

**Figure 1:**
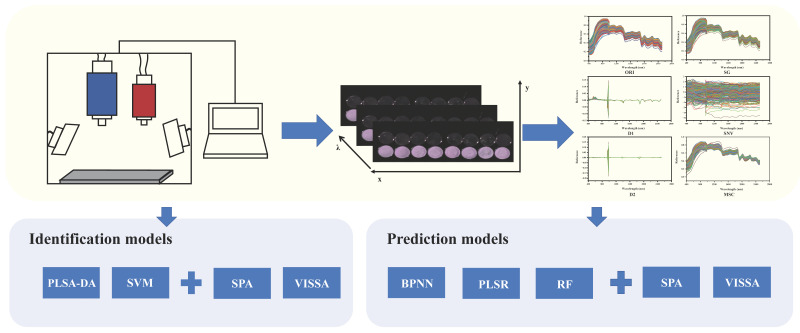
The specific analysis workflow.

In the classification model, 420 samples were divided into a calibration set and a prediction set at a ratio of 7.5:2.5. In the prediction model, the samples were divided into a calibration set and a prediction set at a ratio of 7:3. In this study, curve correlation coefficient (R^2^), root mean squared error (RMSE), and residual predictive deviation (RPD) were taken to evaluate the regression model performance. Specifically, accuracy was used to evaluate the performance of the classification model. When the R^2^ value ranged between 0.60~0.80 and the RPD value ranged between 2.0~2.5, it indicated that the model could be used for indicator prediction. When the R^2^ value ranged between 0.80~0.90 and the RPD value ranged between 2.5~3.0, it indicated that the model had a good prediction effect. When the R^2^ value exceeded 0.90 and the RPD value exceeded 3.0, it indicated that the model had a very good prediction effect [23]. The specific analysis workflow of the study is illustrated in Figure 1.

## 3. Results

### 3.1. Statistical Analysis of Tanshinone Content in S. miltiorrhiza from Different Origins

The contents of the four types of tanshinones in *S. miltiorrhiza* from different origins are listed in Table 1. The result suggested that there were significant differences (*p* < 0.05) in the contents of the four types of tanshinones in *S. miltiorrhiza* of different origins. The content of Tan I ranged from 0.132 mg/g in *S. miltiorrhiza* from Shanxi to 1.090 mg/g in *S. miltiorrhiza* from Shandong. The content of Tan IIA ranged from 0.826 mg/g in *S. miltiorrhiza* from Hebei to 2.807 mg/g in *S. miltiorrhiza* from Shandong. The content of CTS ranged from 0.451 mg/g in *S. miltiorrhiza* from Hebei to 3.616 mg/g in *S. miltiorrhiza* from Shandong. The content of total tanshinone ranged from 1.593 mg/g to 7.469 mg/g, and it was the highest in *S. miltiorrhiza* from Shandong. Meanwhile, the content of total tanshinones in *S. miltiorrhiza* from Anhui was high. The content of total tanshinones in *S. miltiorrhiza* from Hebei was the lowest.

### 3.2. Raw spectra Characteristics of S. miltiorrhiza

**Figure 2:**
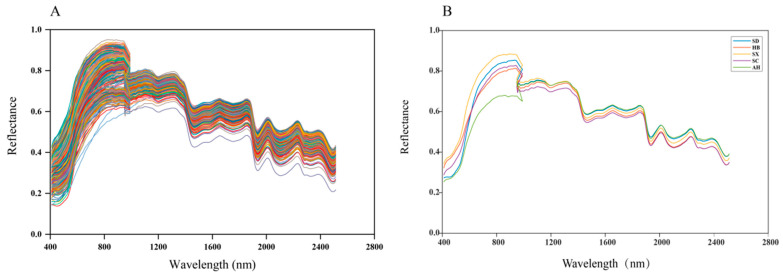
Spectral curves of *S. miltiorrhiza* powder samples of different origins. (**A**) The original spectral curve of 420 samples. Each line with a kind of different color represents a wavelength. And (**B**) the average spectral curves of *S. miltiorrhiza* powder samples from the five origins.

The spectral curves of the *S. miltiorrhiza* powder of different origins exhibited similar trends, but there were some differences in their reflectance intensities (Figure 2A). This may be due to the commonality of the internal tissues of *S. miltiorrhiza* from different origins and the differences in the content of the chemical components. As demonstrated in Figure 2B, the spectral curve showed a sharp upward trend at 400~900 nm. In the range of 900~2800 nm, the spectral curve showed a fluctuating downward trend with obvious peaks and troughs.

The peaks at different wavelengths were analyzed. The average spectral reflectance intensities of *S. miltiorrhiza* powder from different origins exhibited large differences in the range of 400~700 nm in the visible region, which may lead to different colors of the samples. The optimal wavelength range for tanshinones was 1220~2580 nm [36], and the *S. miltiorrhiza* powder samples from Shandong and Anhui had stronger spectral reflectance in this range. This coincided with the phenomenon that the *S. miltiorrhiza* from Shandong and Anhui had a higher content of tanshinones. The peaks at 910 nm and 960 nm can be attributed to the second overtone of the O-H stretching mode of water or carbohydrates [37]. The peaks at 1120 nm and 1300 nm were related to the second stretching overtones of C-H [38]. The peak at 1740 nm was related to the secondary octave of cyclohexene methylidene stretching vibration, and the peak at 2220 nm was related to the combined frequency of benzene ring C-C stretching vibration and C-H stretching vibration [39].

### 3.3. Classification of the Geographical Origins of S. miltiorrhiza Based on Hyperspectral Imaging Full Wavelengths

To remove baseline shifts and correct for scattering effects, the original spectrum (ORI) was processed with five pretreatment methods (D1, D2, SG, MSC, and SNV). Then, PLS-DA and SVM models combined with ORI and the five pretreatment methods were established to discriminate the origins of *S. miltiorrhiza*. The results of the models are presented in Table 2. Generally, the origins of miltiorrhiza could be discriminated using PLS-DA and SVM models, and the PLS-DA models demonstrated better discrimination ability than the SVM models. In the PLS-DA, the discrimination accuracy on the calibration sets and prediction sets was higher than 95%, indicating that PLS-DA models had good discrimination ability for *S. miltiorrhiza* from different origins. Among all types of PLS-DA models, the D1-PLS-DA model obtained the highest discrimination accuracy of 98.97% on the prediction sets. Among all types of SVM models, the D2-SVM model obtained the highest discrimination accuracy of 95.24% on the prediction sets.

**Figure 3:**
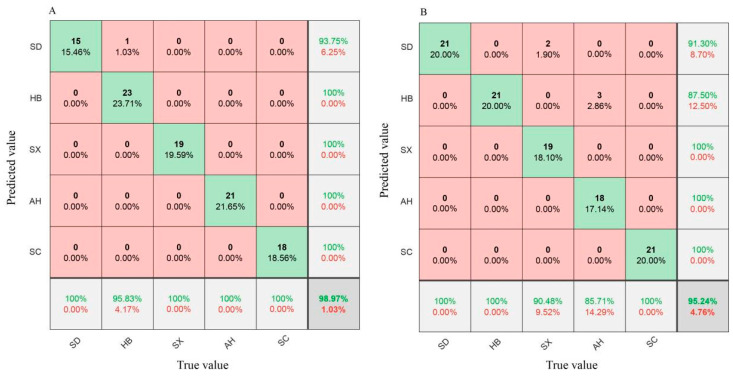
The confusion matrices on the prediction sets of the PLS-DA and SVM models. (**A**) D1-PLS-DA, (**B**) D2-SVM.

To further measure the discrimination ability and discrimination accuracy of PLS-DA and SVM models for *S. miltiorrhiza* of different origins, the optimal models D1-PLS-DA and D2-SVM were selected, and the confusion matrices of their prediction sets were plotted (Figure 3). The results indicated that the D1-PLS-DA model can more accurately discriminate *S. miltiorrhiza* of different origins than the D2-SVM model. In the PLS-DA model, one *S. miltiorrhiza* sample from Hebei was misidentified as a sample from Shandong (Figure 3A). In the SVM model, two *S. miltiorrhiza* samples from Shanxi were misidentified as samples from Shandong, and three *S. miltiorrhiza* samples from Anhui were misidentified as samples from Hebei (Figure 3B).

### 3.4. Prediction of Chemical Indicators Based on HSI Full Wavelength

**Figure 4:**
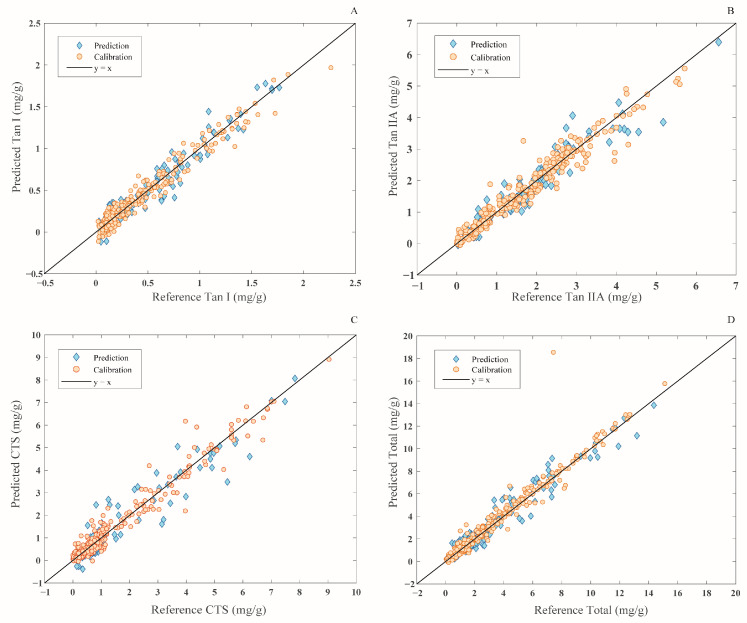
The best-performing prediction model of the four chemical indicators based on the full wavelength. (**A**) Prediction of Tan I by the MSC-PLSR, (**B**) prediction of Tan IIA by the ORI-BPNN, (**C**) prediction of CTS by the SG-BPNN, and (**D**) prediction of the total tanshinones by the SNV-BPNN.

The BPNN, PLSR, and RF were combined with five pretreatment methods (D1, D2, SG, MSC, SNV) and ORI to establish the prediction models. The results of the different models for predicting the four chemical indicators of *S. miltiorrhiza* are presented in Table 3. In the prediction of Tan I, D1-BPNN and D2-BPNN models demonstrated a slight decrease in performance, and the rest of the BPNN and PLSR models obtained good prediction results. Among them, MSC-PLSR (Figure 4A) achieved the best prediction effect. In the prediction of Tan IIA, the ORI-BPNN and MSC-BPNN models obtained good predictive effects, with the ORI-BPNN (Figure 4B) model demonstrating the best prediction effect. In the prediction of CTS, the ORI-BPNN, SG-BPNN, and MSC-BPNN models showed a very good effect. In particular, the SG-BPNN (Figure 4C) model obtained the best prediction effect among them. In the prediction of total tanshinone content, the BPNN and PLSR models combined with ORI, SNV, MSC, and SG obtained excellent prediction results, and the SNV-BPNN (Figure 4D) model obtained the best prediction effect.

Overall, the BPNN and PLSR models showed better prediction capability, and the RF model showed poor prediction capability. It indicated that the RF model was not suitable for predicting the content of tanshinones in *S. miltiorrhiza*. Among the four components, total tanshinone content was the best predicted. This was probably because Tan I, Tan IIA, and CTS were structurally similar. The three components may interfere with each other for the final spectral information produced when the content of a single component is predicted.

### 3.5. Classification and Prediction of Chemical Indicators Based on Selected Wavelengths

#### 3.5.1. Classification of the Geographical Origins of S. miltiorrhiza Based on Selected Wavelengths

**Figure 5:**
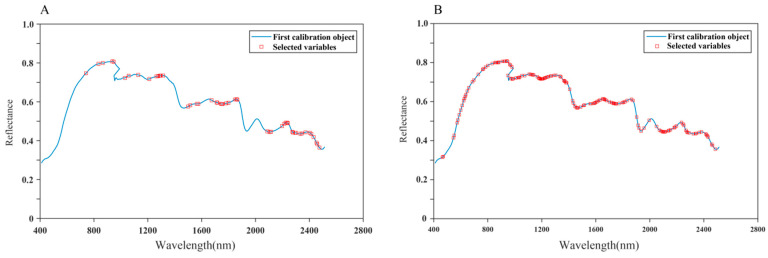
Selected wavelengths for best classification model of *S. miltiorrhiza* by SPA and VISSA. (**A**) SPA-D1-PLS-DA, (**B**) VISSA-D1-PLS-DA.

To obtain the best effective wavelength screening method and further simplify the models, based on the results of the classification models established with full wavelengths, this study extracted the effective wavelengths using the SPA and VISSA methods for the D1-PLS-DA model with the best classification effect. The results are illustrated in Table 4 and Figure 5.

Compared with the full-wavelength models, the performance of the D1-PLS-DA model was improved when the SPA and VISSA were employed. The accuracy on the training and prediction sets all reached 100%. The number of effective wavelengths extracted by the SPA and VISSA was 51 and 150, respectively. It can be found that the number of wavelengths extracted by the SPA is much smaller than that extracted by the VISSA. Further observing the distribution of effective wavelengths, there are 25 same effective wavelengths (Figure 5A,B). This indicated that it may contain information about differences in *S. miltiorrhiza* samples from different origins.

#### 3.5.2. Prediction of Chemical Indicators Based on HSI Selected Wavelengths

Based on the content prediction results at full wavelengths, the best-performing model for the prediction of the four components was selected to extract effective wavelengths, and the results are shown in Table 5. Comparing the number of effective wavelengths obtained by the SPA and VISSA, the number of effective wavelengths extracted by the SPA for CTS, Tan IIA, Tan I, and the total tanshinones were 36, 33, 21, and 33, respectively. The number of wavelengths was reduced by 90.91%, 91.67%, 94.70%, and 91.67%, respectively. The number of effective wavelengths extracted by the VISSA were 100, 104, 131, and 111, respectively. It can be seen that the SPA had less wavelength information and could simplify the model more. Then, the performance of each effective wavelength model was further compared. With the SPA and VISSA, the models demonstrated good performance for the prediction of the four components. The R^2^ values on the prediction sets and the training sets were greater than 0.9, and the RPD values were greater than 3. From the results, the number of effective wavelengths extracted by the SPA was much smaller than that of the VISSA when both contributed to good model performance. This suggested that the SPA was more suitable for extracting effective wavelengths for the prediction of chemical indicators in *S. miltiorrhiza*.

**Figure 6:**
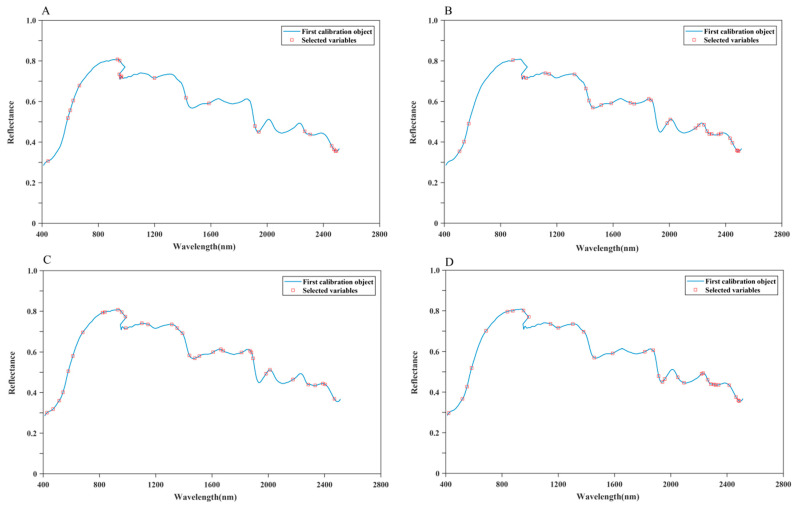
Selected wavelengths for best prediction of the chemical indicators of *S. miltiorrhiza* samples by the SPA. The selected wavelengths are shown in red boxes. (**A**) The elected wavelengths for the prediction of Tan I (MSC-PLSR), (**B**) the selected wavelengths for the prediction of Tan IIA (ORI-BPNN), (**C**) the selected wavelengths for the prediction of CTS (SG-BPNN), and (**D**) the selected wavelengths for the prediction of total tanshinones (SNV-BPNN).

The VISSA obtained eight of the same effective wavelengths for the prediction of the four components, and they were 480, 540, 1035, 1461, 1466, 1499, 1504, and 1510 nm. In comparison, the SPA did obtain the same effective wavelengths, indicating that the effective wavelengths extracted by the SPA are more representative (Figure 6).

## 4. Discussion

In this study, the tanshinone content of *S. miltiorrhiza* from different origins was determined. It was found that the tanshinone content of *S. miltiorrhiza* from Shandong and Anhui was higher, which was consistent with the results obtained by Wang et al. [35]. *S. miltiorrhiza* is a widely distributed species in China. The quality of *S. miltiorrhiza* varies from place to place due to differences in environment, germplasm, and cultivation methods, etc. [40]. Liang et al. found that conditions in terms of soil effective phosphorus and precipitation were more favorable for tanshinone IIA accumulation in *S. miltiorrhiza* in Mengshan, Shandong [2]. Zhang et al. found the specific geospatial distribution of these active ingredients of *S. miltiorrhiza* in China, due to the climatic factors [41]. Zhang et al. found that although *S. miltiorrhiza* from the same region, Ji’an (Jiangxi province), were varied according to soil inconsistence, the climate factors among different regions in a larger spatial scale had more effect on *S. miltiorrhiza* [42]. Therefore, there were differences in the constituent contents of *S. miltiorrhiza* in the same provinces, but the differences in the contents of *S. miltiorrhiza* from different provinces were more significant [43]. The consistency of the quality of *S. miltiorrhiza* was high in Sichuan [44]. The same findings were made for *Coptis* [45], *Sorghum bicolor* (L.) Moench [46], and *Curcuma Species* Grown [47], whose qualities are also differences in different origins. This implies that there is a great market potential for the use of hyperspectral imaging in origin identification.

Sun et al. combined HSI with a SVM to establish a classification model of *S. miltiorrhiza* from different origins and obtained better results [48]. In this study, in addition to the SVM model, the PLS-DA model was employed, and it was found that the PLS-DA model performed better than the SVM model. In the classification models, the performance was improved after ORI was preprocessed, except for the SNV-SVM model, whose performance was degraded. This may be because, for SVM models, SNV preprocessing amplifies the noise in the raw data, leading to a degradation in model performance.

Infrared spectroscopy has been used for the determination of tanshinone content in *S. miltiorrhiza* [49], but there is little research on the use of HSI. In this study, it was found that RF models had poor performance in the prediction of tanshinone content of *S. miltiorrhiza*, with RPD values all less than 2.5. It indicated that RF models were not suitable for the content prediction of *S. miltiorrhiza*. Among the BPNN and PLSR models, the best prediction models for the four types of tanshinones were not the same. This was consistent with the results obtained by Wang et al. [20]. Different model combinations have different effects on model performance. Xia et al. found that the CARS-BP-ANN model had the best performance to predict chlorogenic acid, luteoloside, and 3,5-Odicaffeoylquinic acid in chrysanthemum [50]. He et al., found that PLSR models with full or selected wavelengths obtained good performance to predict SO_2_ residual content in *Fritillaria thunbergii* Bulbus [51]. Li et al., found that the detrending-SPA-BPNN had good model performance to determine N content in citrus leaves [52]. Liu et al. found that the RFOK model had excellent spatial distribution prediction ability for soil heavy metal pollution, compared with the RF model and ordinary kriging model [53]. In the models of CARS, IVSO, or VISSA combined with the PLSR or SVR, Wang et al., found that CARS-SVR exhibited good model performance [54]. Therefore, corresponding models should be selected to predict the composition of samples. However, this is not conducive to developing a miniaturized hyperspectral device that can predict the chemical content of all Chinese medicine. So, in future research, a unified prediction model will be studied in depth.

Combining the effective wavelengths of the classification and prediction models, the same effective wavelengths for the SPA-D1-PLS-DA to predict CTS content were 1313, 2333, and 2403 nm. The same effective wavelengths for the SPA-D1-PLS-DA to predict Tan I were 935, 2458, and 2474 nm. The same effective wavelengths for the SPA-D1-PLS-DA to predict Tan IIA were 1515, 1749, 2300, and 2431 nm. The same effective wavelength for the SPA-D1-PLS-DA to predict total tanshinones was 2414 nm. This suggested that the four types of tanshinones provided part of the information for distinguishing *S. miltiorrhiza* from different origins. Different effective wavelengths were analyzed in correlation with the chemical structures. The peak near 1300 nm was related to the second overtone of C-H stretching [55], and the peak within 2300~2400 nm was related to the combined frequency of methyl stretching and bending vibrations [32,56]. The peak near 2500 nm was related to the combination of C-H stretching and bending, and the peak within 1500~1904 nm was related to the second overtone of C=O stretching and the first overtone of C-H stretching [57].

## 5. Conclusions

The origin and content of tanshinones are important indexes to judge the quality of *S. miltiorrhiza.* The content of tanshinones in *S. miltiorrhiza* from different origins was different. In this study, HSI combined with chemometrics was used to quickly and accurately identify the origin of *S. miltiorrhiza* and predict the content of tanshinone components. The SPA largely simplifies the model in addition to making it perform well. 

The overall results indicate that HSI technology has great potential for the origin traceability and quality evaluation of *S. miltiorrhiza*. Compared with traditional methods, it has the advantages of non-destructive and rapid detection. To further improve the applicability of the model, we will increase the chemical indexes of *S. miltiorrhiza* and expand the collection sources of *S. miltiorrhiza* in future studies. It is expected to provide some references for the development of proprietary HSI equipment to achieve origin traceability and quality evaluation of *S. miltiorrhiza*.

## Figures and Tables

**Table 1:** Content of tanshinones in *S. miltiorrhiza* of different origins.

Collection Origins	Tan I (mg/g)	Tan IIA (mg/g)	CTS (mg/g)	Total (mg/g)
SD (*n* = 84)	1.090 ± 0.373 d	2.807 ± 0.983 d	3.616 ± 2.070 d	7.469 ± 3.126 d
HB (*n* = 84)	0.316 ± 0.268 ab	0.826 ± 0.633 a	0.451 ± 0.379 a	1.593 ± 1.231 a
SX (*n* = 84)	0.132 ± 0.058 c	1.142 ± 0.608 b	0.640 ± 0.420 b	1.914 ± 1.014 a
SC (*n* = 84)	0.271 ± 0.186 a	1.663 ± 1.317 c	0.678 ± 0.687 ab	2.612 ± 2.066 b
AH (*n* = 84)	0.376 ± 0.257 b	1.940 ± 1.113 c	1.928 ± 1.831 c	4.245 ± 2.786 c

The five origins include Shandong (SD), Hebei (HB), Shanxi (SX), Sichuan (SC), and Anhui (AH). Lowercase letters such as a, b, c, etc., indicate a statistical difference (*p* < 0.05) between different letters and no statistical difference between the same letters.

**Table 2:** Discrimination of *S. miltiorrhiza* origins using PLS-DA and SVM models.

Pretreatments	PLS-DA		SVM	
	Calibration Set (%)	Prediction Set (%)	Calibration Set (%)	Prediction Set (%)
ORI	98.45	95.88	98.73	80.95
D1	99.69	98.97	99.37	91.43
D2	98.45	97.94	99.05	95.24
SG	99.07	97.94	98.73	80.95
MSC	99.07	96.91	97.46	85.71
SNV	99.69	97.94	100.00	75.24

**Table 3:** Four chemical indicators prediction based on the full wavelengths.

Chemical Indexes	Models	Calibration Set		Prediction Set			Chemical Indexes	Models	Calibration Set		Prediction Set		
		R^2^	RMSEC	R^2^	RMSEP	RPD			R^2^	RMSEC	R^2^	RMSEP	RPD
Tan I	ORI-BPNN	0.965	0.079	0.924	0.118	3.35	Tan IIA	ORI-BPNN	0.945	0.282	0.917	0.332	3.34
	D1-BPNN	0.948	0.108	0.861	0.163	2.53		D1-BPNN	0.949	0.275	0.871	0.431	2.63
	D2-BPNN	0.950	0.095	0.846	0.172	2.51		D2-BPNN	0.899	0.384	0.820	0.503	2.33
	SG-BPNN	0.966	0.079	0.917	0.125	3.18		SG-BPNN	0.918	0.348	0.885	0.391	2.66
	MSC-BPNN	0.971	0.071	0.919	0.121	3.41		MSC-BPNN	0.939	0.297	0.919	0.328	3.28
	SNV-BPNN	0.961	0.083	0.930	0.114	3.44		SNV-BPNN	0.957	0.258	0.886	0.393	2.81
	ORI-PLSR	0.960	0.081	0.934	0.117	3.90		ORI-PLSR	0.940	0.296	0.864	0.422	2.66
	D1-PLSR	0.950	0.091	0.906	0.136	3.24		D1-PLSR	0.946	0.281	0.821	0.489	2.32
	D2-PLSR	0.940	0.100	0.910	0.132	3.25		D2-PLSR	0.895	0.391	0.826	0.478	2.32
	SG-PLSR	0.970	0.072	0.931	0.123	3.79		SG-PLSR	0.935	0.308	0.851	0.442	2.51
	MSC-PLSR	0.955	0.087	0.938	0.110	4.03		MSC-PLSR	0.950	0.269	0.853	0.439	2.53
	SNV-PLSR	0.976	0.063	0.932	0.120	3.83		SNV-PLSR	0.932	0.316	0.841	0.455	2.39
	ORI-RF	0.9600	0.119	0.880	0.218	1.64		ORI-RF	0.946	0.400	0.860	0.578	1.78
	D1-RF	0.9795	0.084	0.923	0.175	2.16		D1-RF	0.974	0.291	0.919	0.448	2.21
	D2-RF	0.9761	0.099	0.928	0.173	2.06		D2-RF	0.955	0.390	0.915	0.472	1.93
	SG-RF	0.9622	0.117	0.868	0.227	1.59		SG-RF	0.952	0.385	0.853	0.592	1.73
	MSC-RF	0.9641	0.110	0.891	0.211	1.77		MSC-RF	0.952	0.373	0.867	0.572	1.91
	SNV-RF	0.9690	0.102	0.902	0.196	2.03		SNV-RF	0.959	0.354	0.902	0.485	2.18
CTS	ORI-BPNN	0.966	0.327	0.911	0.534	3.03	Total (Tan I + Tan IIA + CTS)	ORI-BPNN	0.956	0.658	0.933	0.803	3.83
	D1-BPNN	0.881	0.612	0.860	0.659	2.56		D1-BPNN	0.939	0.778	0.886	1.106	2.96
	D2-BPNN	0.897	0.563	0.711	0.966	1.68		D2-BPNN	0.939	0.758	0.803	1.384	2.15
	SG-BPNN	0.927	0.483	0.911	0.527	3.25		SG-BPNN	0.917	0.888	0.920	0.880	3.24
	MSC-BPNN	0.881	0.607	0.913	0.529	3.01		MSC-BPNN	0.951	0.699	0.927	0.856	3.41
	SNV-BPNN	0.926	0.480	0.907	0.556	2.79		SNV-BPNN	0.943	0.794	0.940	0.759	4.01
	ORI-PLSR	0.927	0.455	0.830	0.783	2.25		ORI-PLSR	0.934	0.770	0.929	0.866	3.53
	D1-PLSR	0.901	0.530	0.770	0.914	1.92		D1-PLSR	0.967	0.547	0.864	1.206	2.66
	D2-PLSR	0.897	0.538	0.711	0.911	1.75		D2-PLSR	0.940	0.733	0.859	1.216	2.44
	SG-PLSR	0.927	0.582	0.911	0.718	2.39		SG-PLSR	0.945	0.705	0.919	0.927	3.32
	MSC-PLSR	0.907	0.513	0.817	0.811	2.14		MSC-PLSR	0.931	0.787	0.909	0.985	3.02
	SNV-PLSR	0.903	0.525	0.823	0.798	2.17		SNV-PLSR	0.949	0.675	0.907	0.986	3.11
	ORI-RF	0.950	0.550	0.878	0.956	1.50		ORI-RF	0.971	0.738	0.912	1.369	1.99
	D1-RF	0.968	0.441	0.894	0.890	1.63		D1-RF	0.977	0.948	0.906	1.374	2.03
	D2-RF	0.960	0.494	0.904	0.842	1.78		D2-RF	0.977	0.687	0.927	1.275	2.05
	SG-RF	0.949	0.542	0.876	0.947	1.56		SG-RF	0.963	0.828	0.907	1.418	1.89
	MSC-RF	0.953	0.519	0.888	0.879	1.83		MSC-RF	0.971	0.717	0.917	1.287	2.30
	SNV-RF	0.958	0.495	0.872	0.930	1.73		SNV-RF	0.970	0.748	0.913	1.343	2.06

**Table 4:** The classification results of the D1-PLS-DA model based on effective wavelengths.

Model	Methods	LV	Number	Calibration Set (%)	Prediction Set (%)
D1-PLS-DA	SPA	5	51	100	100
	VISSA	13	150	100	100

**Table 5:** The prediction of four chemical indicators based on the effective wavelengths selected.

Chemical	Model	Method	Wavelengths Number	Calibration Set		Prediction Set		
				R^2^	RMSEC	R^2^	RMSEP	RPD
Tan I	MSC-PLSR	SPA	21	0.931	0.107	0.937	0.109	3.94
		VISSA	131	0.955	0.087	0.940	0.113	4.08
Tan IIA	ORI-BPNN	SPA	33	0.906	0.369	0.905	0.357	3.17
		VISSA	104	0.952	0.263	0.902	0.362	3.12
CTS	SG-BPNN	SPA	36	0.962	0.345	0.910	0.528	3.24
		VISSA	100	0.931	0.476	0.902	0.560	3.62
Total	SNV-BPNN	SPA	33	0.941	0.759	0.933	0.830	3.44
		VISSA	111	0.959	0.626	0.931	0.830	3.58

## Data Availability

The original contributions presented in the study are included in the article/Supplementary Materials; further inquiries can be directed to the corresponding author.

## Supplementary Materials

The following supporting information can be downloaded at: https://www.mdpi.com/article/10.3390/foods13223673/s1, Table S1: Information for the sampling locations of *Salvia miltiorrhiza*, Figure S1: Total ion current diagrams of a Salvia miltiorrhiza sample: 1: tanshinone I 2: cryptotanshinone, 3: tanshinone IIA.
