# Supplementary material for: Tanshinone Content Prediction and Geographical Origin Classification of Salvia miltiorrhiza by Combining Hyperspectral Imaging with Chemometrics
Source: Foods. 2024 Nov 18;13(22):3673. doi: 10.3390/foods13223673 (PMC11593691; doi:10.3390/foods13223673)
Supplement: Supplementary file 1 [file foods-13-03673-s001.zip › foods-3280153-supplementary.pdf]

Table S1 Information for the sampling locations of *Salvia miltiorrhiza*

No.	Locations	Longitude (°E)	Latitude (°N)	Altitude (m)	Time
S1~14	Pingyi, Shandong	117.62	35.35	257	2021-10
S15~28	Pingyi, Shandong	117.75	35.57	228	2021-10
S29~42	Pingyi, Shandong	117.92	35.90	227	2021-10
S43~56	Zhencheng, Shandong	115.40	35.58	44	2021-11
S57~70	Zhencheng, Shandong	115.49	35.50	41	2021-11
S71~84	Yuncheng, Shandong	115.68	35.66	47	2021-11
S85~98	Lingshou, Hebei	114.43	38.33	108	2021-10
S99~112	Lingshou, Hebei	114.43	38.34	104	2021-10
S113~126	Lingshou, Hebei	114.29	38.51	102	2021-10
S127~140	Anguo, Hebei	115.25	38.48	29	2021-10
S141~154	Anguo, Hebei	115.25	38.45	28	2021-11
S155~168	Anguo, Hebei	115.30	38.34	NA	2021-11
S169~182	Ruicheng, Shanxi	110.59	34.72	653	2021-11
S183~196	Wanrong, Shanxi	110.87	35.37	675	2021-11
S197~210	Wanrong, Shanxi	110.87	35.37	675	2021-11
S211~224	Wanrong, Shanxi	110.59	35.39	502	2021-11
S225~238	Hejin, Shanxi	110.59	35.54	368	2021-11
S239~252	Quwo, Shanxi	111.59	35.62	554	2021-11
S253~266	Zhongjiang, Sichuan	104.61	30.93	449	2021-11
S267~280	Zhongjiang, Sichuan	104.59	30.99	737	2021-11
S281~294	Zhongjiang, Sichuan	104.51	30.99	753	2021-12
S295~308	Bazhou, Sichuan	106.81	31.90	555	2021-12
S308~322	Bazhou, Sichuan	107.02	31.96	717	2021-12
S323~336	Bazhou, Sichuan	107.01	31.97	659	2021-12
S337~350	Qiaocheng, Anhui	115.65	33.86	45	2021-11
S351~364	Qiaocheng, Anhui	115.67	33.77	34	2021-11
S365~378	Qiaocheng, Anhui	115.65	33.74	30	2021-11
S379~392	Qiaocheng, Anhui	115.88	33.83	27	2021-11
S393~406	Mengcheng, Anhui	116.74	33.27	23	2021-11
S407~420	Mengcheng, Anhui	116.77	33.04	18	2021-11

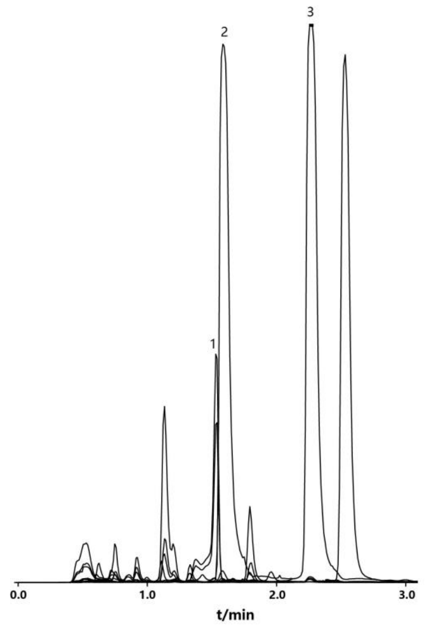

Figure S1 Total ion current diagrams of a *Salvia miltiorrhiza* sample

1:tanshinone I 2:cryptotanshinone 3：tanshinone IIA
